# Preservation of Pathological or Other Specimens

**Published:** 1904-04-23

**Authors:** 


### PRESERVATION OF PATHOLOGICAL OR OTHER SPECIMENS.

The practitioner very often wishes to preserve a specimen of some diseased organ or tissue, either for museum purposes, or for reference, or to show at societies. The two most useful methods available for this purpose are (1) The spirit method, and (2) the formalin method. The first one, which held the field until recent years, has now been almost given up in preference for the second. The disadvantage of using methylated spirit for the preservation of museum specimens is that the original colours, as seen in the recent condition, after a short time fade, and, therefore, much of the usefulness of the specimen is lost. The advantage of the spirit method is that it is fairly cheap and much less troublesome than the formalin method. It is generally recognised now, however, that the latter is the best and most satisfactory in spite of the extra trouble and expense which it entails, for the results are excellent, and, with a little care and attention to details, very useful and pleasing specimens may be prepared. A short description of the method employed in using spirit is, however, appended, in addition to a fuller account of the formalin process.

*Spirit Method.*—Wash the specimen well in a large quantity of water—running water is best if available—to get rid of as much blood as possible; then place it in a vessel containing a mixture of ordinary methylated spirit and water in equal parts. Cotton-wool may be used to keep the specimen off the bottom of the jar and from touching the sides, and, if it is a hollow organ, it is better to stuff the specimen lightly with wool. By this means the natural shape of the specimen is preserved. As a rule there is much discoloration of the spirit by the escape of blood from the specimen at the end of 24 hours, so it is advisable to stir up the bottom layer of fluid, or better still, replace it by a fresh solution. A specimen preserved in this way will keep indefinitely with an occasional change of spirit.

*Formalin Method.*—The following are the solutions required and directions for carrying out the process:—

| | | |
|---|---|---|
| *A.* Formalin solution : | Pure formalin | 150 cc. |
| | Pot. nitrate | 15 grm. |
| | Pot. acetate | 30 grm. |
| | Tap water | 1,000 cc. |
| *B.* Methylated spirit (strong). | | |
| *C.* Glycerin solution : | Glycerin | 1,000 cc. |
| | Pot. acetate | 30 grm. |
| | Distilled water | 1,200 cc. |
| | Pure formalin | 20 cc. |

Dissolve the potassium acetate in some of the distilled water and filter it, then mix the whole of the ingredients together and the solution is ready for use. Place the organ or tissue to be preserved into a large jar or basin containing some of the solution *A*, taking care to arrange the specimen in its natural position or shape, after first rinsing any loose blood off its surface. Ordinary absorbent cotton wool is the best material to use for supporting and packing round the specimen; this keeps the latter from touching the sides or bottom of the jar and so being flattened out of shape by pressure. In the case of a hollow viscus such as the stomach or a piece of intestine, it is better, when possible, to distend the specimen with formalin solution and tie the ends; this is best done while the specimen is half immersed in the fluid in the jar. The specimen may also be supported by threads passing from it to the sides of the vessel. As formalin hardens the skin of the hands very quickly, it is as well to use a pair of indiarubber gloves if these are available, and in any case forceps will save the hands a good deal. After soaking in the formalin solution for a short time, it will be noticed that the colour of the specimen changes to a dirty grey; this, however, does not matter, because most of the colour will return in the next solutions. The specimen soon becomes hard and fixed, so that the "shaping" of the parts with the wool packing should be done as quickly as possible. The length of time which it is necessary to leave specimens in the formalin solution varies according to their size and consistence. A small and delicate specimen, as for instance a piece of intestine should only be left in for about 15–20 hours, whereas a thick piece of liver or lung will require 48 hours, and it will not harm then if left in for a longer period. The specimen is next transferred to solution *B*; here, if the specimen is small and delicate about 10 to 20 minutes is quite long enough to leave it in the spirit, and it will be noticed that within a few minutes the original colour begins to return; as soon as there is any signs of the colour fading while the specimen is in the spirit it must be at once taken out. Large specimens may be left in the spirit for an hour or so.

The next step is the placing of the specimen in the last solution, solution *C.*, where it will be found that the colour will return, in most cases to what it was in the fresh condition of the specimen. It takes some time for a solid specimen like a piece of liver or kidney to sink in the glycerin solution, so that it is as well to place some cotton wool lightly on its surface to keep it moist. Lung and fatty tissues will not sink and so must be weighted if it is intended to mount the specimens. Specimens may be kept permanently in the glycerin solution, the only precautions necessary being the occasional addition of a small quantity of formalin to prevent growth of moulds, and if the solution become much discoloured it must be changed. Some form of covering is necessary for the vessels used, either a glass sheet or stopper.

